# Evaluation of Antimalarial Activity of the Leaf Latex and TLC Isolates from* Aloe megalacantha* Baker in* Plasmodium berghei* Infected Mice

**DOI:** 10.1155/2019/6459498

**Published:** 2019-04-14

**Authors:** Gebretsadkan Hintsa, Gereziher Geremedhin Sibhat, Aman Karim

**Affiliations:** Department of Pharmacognosy, School of Pharmacy, College of Health Sciences, Mekelle University, P.O. Box 1871, Mekelle, Ethiopia

## Abstract

Malaria is a devastating parasitic disease which caused around 216 million cases and 445,000 deaths worldwide in 2016. This might be attributed to a wide spread of drug resistant parasites. The plant* Aloe megalacantha* is indigenous to Ethiopia where the sap of the leaves is traditionally used for the treatment of malaria. This study was aimed at evaluating the antimalarial effect of leaf latex and isolates obtained from* Aloe megalacantha* against chloroquine sensitive* Plasmodium berghei *ANKA strain in Swiss albino mice. Peters' 4-day suppressive test method was used to test the antimalarial activity of both leaves latex and isolates. Three isolates were obtained using thin layer chromatography and were coded as AM_1_, AM_2_, and AM_3_ in ascending order of their retention factor. After treatment of* Plasmodium berghei* infected mice with leaf latex of* Aloe megalacantha* for four days at 100, 200, and 400 mg/kg, it shows 30.3%, 43.4%, and 56.4% suppression of the parasite growth, respectively. 32.3%, 51.3%, and 67.4% chemosuppression after treatment with AM_1_, 39.8%, 50.6%, and 64.2% chemosuppression after treatment with AM_2_, and 52.6%, 69.4%, and 79.6% chemosuppression after treatment with AM_3_ were observed at doses of 100, 200, and 400 mg/kg/day, respectively. The observed parasite suppression of leaves latex and isolates was statistically significant (P<0.05) as compared to negative control. Moreover, both the leaves latex and isolates were also observed to prevent* Plasmodium berghei *induced body weight loss and hypothermia and increased the survival time of* Plasmodium berghei* infected mice as compared to the negative control. Hence, the present study supports the traditional claim of the plant for the treatment of malaria.

## 1. Background

Malaria is the most devastating infectious disease caused by the protozoan parasites belonging to the genus* Plasmodium*. There are five parasite species that cause malaria in humans, namely,* Plasmodium falciparum*,* Plasmodium vivax*,* Plasmodium malariae*,* Plasmodium ovale*, and* Plasmodium knowlesi*, with* P. falciparum *causing the most severe form of malaria and hence higher mortality rates [1]. Despite the progress in reducing malaria cases and deaths, beyond half of the total population are still at risk of infection with malaria. Malaria has been reported to cause 216 million cases and 445,000 deaths globally in 2016. From these, approximately 90% of the cases and 91% of the deaths were in the African region. It has reported that about 70% of the deaths were in children less than 5 years of age [2]. In addition to the suffering of individuals due to malaria, it weakens the economy of developing countries and it is the leading causes of outpatient visits and hospital admission because of different reasons such as limited number of antimalarial drugs, rapid emergence of drug resistant strains of parasite, and emergence of new* Plasmodium* species, particularly* P. knowlesi* [3]. These problems encouraged the urgent need to develop new affordable and safe antimalarials with novel modes of action. One approach to this is searching novel antimalarial drugs from traditionally acclaimed medicinal plants [4, 5].

Nearly 80% of population of the world still rely heavily on traditional healers and need medicinal plants for treatment of various kinds of ailments. Traditional medicines are often more available, affordable, and sometimes are perceived as more effective than modern drugs [6, 7]. Medicinal plants most frequently used in Ethiopia and neighboring countries as traditional medicine for the treatment of malaria include* Acalypha fruticosa*,* Azadirachta indica*,* Dendrosicyos socotrana *[6],* Ajuga integrifolia, Melia azedarach, Peponium vogelii, Premna schimperi, Clerodendrum myricoides, Croton macrostachyus, Fagaropsis angolensis *[8],* Dodonaea angustifolia, Aloe debrana *[9],* Aloe *species,* Azadirachta indica*,* Tamarindus indica *[8, 10, 11],* Gardenia ternifolia*,* Indigofera spicata *[12], and* Combretum molle *[13].

*Aloes *especially* A. vera *have a very long history of medicinal, cosmetic, and cultural uses. There are about 600 different* Aloe* species recognized so far; most of them are native to South Africa, the Arabian Peninsula, Madagascar, and other islands of the western Indian Ocean [14]. Ethiopia has 46 species of* Aloe*, of these* Aloe megalacantha* is indigenous to Ethiopia and rarely found in Northern Somalia [15, 16].

The leaf latex of* Aloe* species is used particularly for the treatment of malaria, bacterial infections, fungal infections, parasitic diseases, gastrointestinal disorders, and inflammations [14, 17]. In Northern and Eastern Ethiopia, people use leaf latex of* A. megalacantha* Baker as a folklore medicine for the treatment of different illnesses including malaria, wound, constipation, abdominal pain, impotence, urine retention, snake bite, and amoebiasis [7, 18–20]. However, the traditional use of* A. megalacantha* for malaria treatment is not yet scientifically validated.

Previous* in vivo* and* in vitro* studies have shown* Aloe* species possessing strong antimalarial activity.* In vitro* antiplasmodial activity of leaf extracts and isolated compounds obtained from* A. vera *has been tested using chloroquine sensitive* P. falciparum *and reported showing a dose*-*dependent chemosuppresive effect [21]. Similar studies to assess antimalarial activity using 4-day suppressive test method in* P. berghei* infected mice model of other* Aloe* species have also been conducted which include studies on methanolic leaf extract of* A. debrana* [9] and leaf latex and isolated compounds of* A. percrassa* [22],* A. citrana* [23], and* A. pulcherrima* [24].

Leaf latex of* A. megalacantha* has shown a significant wound healing activity in both excision and incision model. It has also possessed good anti-inflammatory effect in carrageenan induced paw edema model [25]. Ethyl acetate root extract and isolated natural products of* A. megalacantha *have been evaluated for their cytotoxic activities against a human cervix carcinoma cell line KB-3-1 with cryptophycin-52 (IC_50_ = 1.3 × 10^−5^ *μ*M) and griseofulvin (IC_50_ = 19.0 *μ*M) as a standard drugs. The study has been conducted on the basis of its traditional use for wound treatment. The isolates have exhibited good cytotoxic activity with aloesaponarin II (IC_50_ = 0.98 *μ*M) possessing the highest activity as compared to the other isolates and the standard drug griseofulvin [26].

Hence, the present study was conducted to evaluate the antimalarial activity of leaves latex and thin layer chromatography (TLC) isolates from* A. megalacantha* using 4-day suppressive test method in an experimental animal model. The present study was also done to justify the traditional use of this plant product. This study may provide baseline information for the scientific community about the chemosuppressive effect of the specific traditional medicine.

## 2. Materials and Methods

### 2.1. Plant Material

The leaves latex of* Aloe megalacantha* was collected from Kilte Awulaelo district locality called Genfel which is located at 47.7 km north of the regional city, Mekelle, Northern Ethiopia. The plant material was authenticated by Professor Sebsebe Demissew, Department of Biology, Addis Ababa University, and a voucher number GH001 of the specimen was deposited in National herbarium, Department of Biology, Addis Ababa University.

### 2.2. Instruments, Chemicals, Reagents, and Drugs

The chemicals, reagents, and drugs listed below were used to perform the experiments: chloroform with batch number V3M521074A (CALRO EBRA reagents, France;), methanol with batch number V4H4376174I (CALRO EBRA reagents, France), silica gel type G with batch number 126K0028 (Sigma-Aldrich, USA), Giemsa stain, immersion oil, tween 80, chloroquine phosphate (Addis Pharmaceutical Factory PLC, Adigrat, Ethiopia), sodium citrate, distilled water, and normal saline (0.9%). All the chemicals and reagents used were analytical grade and purchased from market.

Drying oven (GENLAB WIDNES, England), TLC glass plate, TLC jar (CAMAG, Germany), Automatic TLC coater (CAMAG, Germany), UV cabinet (CAMAG, Germany), Compound Microscope (Olympus, Germany), oral gavages, stop watch, electronic weighing balance (Adventurer OHAUS, China), and digital thermometer were also used to conduct the study.

### 2.3. Experimental Animals and* Plasmodium berghei*

Swiss albino mice of male sex, weighing 20-25 g and aged of 6-8 weeks, were used to study the antimalarial activity and female Swiss albino mice weighing 25-30 g with 8-12 weeks of age were used to conduct acute oral toxicity test. The experimental mice were obtained from the animal house of School of Pharmacy, Mekelle University, Ethiopia. They were fed with pellet and water* ad libitum*. The mice were used as per the international guideline for care and use of the experimental animals. These mice were housed in a standardized room which has an artificial lighting 12 hr per day to acclimatize them to the laboratory environment [27]. The mice used on the study were euthanized at the end of follow-up period using a chemical method (Halothane in a desiccator). Chloroquine sensitive* Plasmodium berghei* ANKA strain was brought from Ethiopian Public Health Institute (EPHI) and maintained by serial passage of blood from infected mice (donor mice) to the noninfected ones on weekly basis.

### 2.4. Extract Preparation

The leaf latex was extracted and prepared as per the procedure described by Geremedhin* et al*. The leaves of* A. megalacantha* were cut transversally near the base and then inclined in a plastic material to collect the yellow sap from inside of the leaf. Finally, the collected sap was left in shaded open air for two to three days by flooding in wide spaced plastic material to make a thin film by which suiting the evaporation of water content of the sap which then resulted in golden latex [22].

### 2.5. Isolation of Compounds

#### 2.5.1. Preparative Thin Layer Chromatography (PTLC)

Isolation commenced after antimalarial activity of the leaf latex of* A. megalacantha* was established. Isolates were obtained from leaves latex using preparative thin layer chromatography (TLC) prepared by coating the glass plate with silica gel type G (Size: 10-40 *μ*, Sigma-Aldrich, USA) at 0.5 mm thickness in the laboratory of Pharmacognosy department with the automatic TLC coater (CAMAG, Germany). The dried leaves latex was dissolved in methanol and applied directly as a band to the coated preparative TLC (20 cm × 20 cm) over one side of the plate. Chloroform and methanol mixture (4:1) were used as a solvent system to develop the chromatogram [22, 24, 28]. The isolates were checked for their purity using silica gel over TLC plate (0.25 mm thickness).

#### 2.5.2. Visualization

The chromatographic zones were visualized first in daylight and then by using ultraviolet light of wave length 254 and 366 nm in UV lamp. After visualization the chromatographic zones were coded based on ascending order of retention factor (R_*f*_) values. Then, each band was carefully scrapped off separately from the plate and dissolved in methanol and chloroform (1:1), filtered, and concentrated.

### 2.6. Acute Oral Toxicity Test

Nulliparous and nonpregnant female Swiss albino mice were used for acute oral toxicity study. The study was conducted as per the internationally accepted protocol drawn under Organization for Economic Cooperation and Development (OECD) guidelines 425 [29]. Limit test protocol of the OECD guideline was followed for this experiment as similar* Aloe* species have been reported till the dose of 2000 mg/kg [22–25]. A total of five mice were used for the leaf latex of* A. megalacantha *and deprived from food for 3 hrs before administration and 1 hr after administration of the test sample. One mouse was administered leaf latex orally at a dose of 2000 mg/kg by using oral gavage and observed for 48 hrs for any toxicity signs. After 48 hrs, four additional mice were administered with the leaf latex at a dose of 2000 mg/kg. After the administration of the leaf latex, every mouse was observed continuously for the first 30 min; intermittently over a period of 24 hrs; and daily for 14 days for the presence of changes in skin and fur, eye and mucous membrane secretions, tremors, convulsions, diarrhea, lethargy, sleep, and coma. Body weight of each mouse was recorded on the first day, seventh day, and fourteenth day [29].

The acute oral toxicity studies of the TLC isolates were conducted using the above protocol used for leaf latex. All test samples were dissolved in distilled water except the third isolate (AM_3_) which was dissolved in 3% tween 80.

### 2.7. *In Vivo *Antimalarial Activity Test

#### 2.7.1. Preparation of the Inoculums

The parasites were maintained by serial passage of blood from infected mice (donor mice) to the noninfected ones on weekly basis. Blood sample, which was taken from donor mouse with the growing parasitaemia of 20-30%, was diluted using normal saline (0.9 %) to prepare an approximate of 5x10^7^ infected erythrocytes per milliliter of blood suspension. So, each 0.2 ml of blood suspension contains approximately 10^7^ infected erythrocytes, which were the standard inoculums used to infect each experimental animals intraperitoneally (ip).

#### 2.7.2. Antimalarial Activity Test

The* in vivo* antimalarial activity of the leaves latex and isolates was evaluated by the method of Peter's 4-day suppressive test on* Plasmodium berghei* infected Swiss albino mice [30]. The mice were housed in standard transparent cages and maintained on pellet and water* ad libitum* for 7 days to acclimatize the laboratory environment. 25 male mice of 6-8-week age and weighing 20-25 g were infected with 0.2 ml blood suspension (about 1x10^7^ parasitized RBC) intraperitoneally and randomly divided into five groups of five mice per group with three experimental groups and two control groups (one for chloroquine as a positive control and the other distilled water or vehicle as a negative control) for each test sample.

The leaf latex was prepared at three different doses of 100, 200, and 400 mg/kg of body weight of mice and chloroquine at 25 mg/kg in a volume of 1 ml/100 g body weight of the mice. The leaf latex or the standard was administered as a single dose per day and given through oral route using standard oral gavage. Treatment was started 3 h after infection on day 0 and was then continued daily for four days (i.e., from day 0 to day 3) in order of their infection time. On the fifth day (D_4_), thin smears of blood films were obtained from the tail of each mouse and smeared on microscope from the first infected mice first and then in order of their infection time. Then, the smears were fixed with absolute methanol and stained with 10% Giemsa solution for 15 min. The chemosuppression effect of each TLC isolates was tested using the above-mentioned method i.e., 4-day suppressive test method which was applied to test the antimalarial activity of leaf latex. Parasitemia level was determined by counting the number of parasitized erythrocytes out of three random fields of the microscope from each slide. Average percent parasitemia and suppression were calculated using the following formula [22].

(1) %  Parasitemia=Number  of  parasitized  RBCTotal  number  of  RBC  count×100%  Suppression=Mean  parasitemia  of  negative  control−Mean  parasitemia  of  treatmentMean  parasitemia  of  negative  control×100

#### 2.7.3. Determination of Body Weight and Temperature

Body weight [22, 24] and rectal temperature [31] of each mouse in all groups were measured before infection (day 0) and on day 4 using electronic balance and digital thermometer, respectively, to observe the effect of the test sample in body weight and temperature of each mouse in all groups.

#### 2.7.4. Determination of Mean Survival Time

Mortality was monitored daily and the number of days from the time of inoculation of the parasite up to death was recorded for each mouse in the treatment and control groups throughout the follow-up period of 28 days (D_0_-D_27_) for all test samples [22, 27]. The mean survival time (MST) for each group was then calculated using the following formula:

(2) MST=Sum  of  the  survival  time  of  mice  in  a  group daysThe  total  number  of  mice  in  that  group

### 2.8. Data Analysis

Results were analyzed by using software SPSS version 21 and reported as a mean ± standard error of mean (M ± SEM). The one-way analysis of variance (ANOVA) followed by Tukey HSD* post hoc *test was used to compare results among and within groups. The results were considered significant when P<0.05.

## 3. Results

### 3.1. Isolation of Compounds from Leaf Latex of* Aloe megalacantha*

Three yellow amorphous solid isolates were isolated from leaves latex of* Aloe megalacantha* with* R*_f_ values of 0.33, 0.49, and 0.62 using repeated preparative TLC coated with silica gel in chloroform/methanol (4:1) solvent system. The isolates were coded as AM_1,_ AM_2,_ and AM_3_ in ascending order of their *R*_*f*_ values. The isolates appeared as bright yellow when viewed under day light, seen as dark spot under UV light of 254 nm and striking yellow under UV light of 366 nm.

### 3.2. Oral Acute Toxicity Test

The oral acute toxicity study showed that neither mortality nor signs and symptoms of toxicity were caused during the follow-up period after oral administration of the leaves latex or TLC isolates obtained from* A. megalacantha* at a dose of 2000 mg/kg. It was also shown that there was no significant weight change after the administration of each test sample.

### 3.3. *In Vivo *Antimalarial Activity

Chemosuppression effect against* P. berghei* in mice was observed after testing the leaves latex and then after TLC isolates (AM_1_, AM_2_, and AM_3_) obtained from leaves latex of* A. megalacantha *using Peter's 4-day suppressive test method. Among the tested samples, as shown in Table 1, the highest inhibition of parasitemia growth was recorded after administration of AM_3_ at a dose of 400 mg/kg body weight of mice. The result also showed that all the tested samples have dose-dependent antiplasmodial activity; i.e., enhanced suppression of parasitemia growth was observed with increasing the dose of the latex or either of the isolates. Thus, after the administration of the leaves latex, AM_1_, AM_2_, and AM_3_ for 4 days at doses of 400 mg/kg/day, they caused 56.4%, 67.4%, 64.2%, and 79.6% suppression of parasite growth, respectively, with the corresponding vehicle treated mice which got 47.0 ± 3.49% percentage parasitemia. The parasite growth inhibitions observed after treatment with latex, AM_1_, AM_2,_ or AM_3_ at three different doses were statistically significant (P<0.001) when compared to the vehicle treated group (negative control). Chloroquine was observed to absolve the* P. berghei* infected mice from the parasite on the fifth day of the infection time which was significant (P<0.001) when compared to the negative control group. There was also statistically significant difference in the growth inhibition activity of chloroquine as compared with leaves latex and TLC isolates excluding AM_3_ at the highest dose.

Mean survival time (MST) of* P. berghei* infected mice was also recorded to evaluate an antimalarial activity of test substance. The mice treated with leaves latex and isolates of* A. megalacantha *at three different doses were observed to survive for longer time than the negative control group in a dose-dependent manner [Table 1]. AM_3_ treated group of mice were survived for longer time as compared with latex and other isolates, which were alive for 10.4 ± 0.51 days after treatment with at a dose of 400 mg/kg body weight with the corresponding negative control lived for 6.4 ± 0.25 days. Generally, the MST of the highest and higher doses of leaves latex, AM_1_ and AM_2_, and all doses of AM_3_ treated groups were statistically significant (P<0.05) when compared with distilled water treated group. Even though the MST obtained by latex, AM_1_ and AM_2_, at a dose of 100 mg/kg was statistically insignificant (P>0.05) when compared to negative control group, the mice were survived slightly longer time than negative control group. The chloroquine treated mice were alive beyond the follow-up period.

Another parameter that helps to identify antimalarial activity of the leaves latex is prevention of body weight loss in day 4 from day 0. Although infection of mice with* P. berghei* caused about 4.6% body weight loss (as observed in the negative control), treating the mice with both the latex and TLC isolates was shielded from curtail of their body weight [Table 2]. All doses of both leaves latex and isolates were shown a dose-dependent weight gain in* P. berghei* infected mice except leaves latex treated mice at a dose of 100 mg/kg/day, which was observed to cause 0.4% of body weight loss. AM_3_ showed a higher weight gain relative to the standard drug, although the isolate did not eliminate the parasite completely. Therefore, the compound might have an ability to increase in body weight by different mechanisms. Negative control group was noticed with 4.6% weight depreciation with the positive standard group gaining weight by 5.9%.

Leaves latex treated mice at doses of 100, 200, and 400 mg/kg were observed to reduce their rectal temperature on fifth day of after infection by 6.6%, 4.6%, and 3.7%, respectively, while the negative control mice got hypothermic by 8.5% (lost 3.2°C) [Table 3]. Moreover, the isolates were pointed out to prevent body temperature depreciation. As compared to the temperature loss of the negative control group, the isolate (AM_1_, AM_2_, or AM_3_) treated mice were seen to improve their body temperature at doses of 100, 200, and 400 mg/kg body weight of mice in a dose-dependent manner. Chloroquine given mice were detected to prevent hypothermia caused due to the disease as the drug completely destructs the parasite from the blood of mice. At the first day before infection, mean rectal temperature of mice treated with leaf latex, isolates, or chloroquine was statistically not significant (P>0.05) as compared to negative control, conversely the observed prevention in rectal temperature loss after four day treatment with leaves latex, either of the isolates or of chloroquine, was statistically significant (P<0.001) as compared to negative control.

## 4. Discussion

The methanolic extract of leaves of* A. debrana* has shown a dose-dependent inhibition of parasitemia against* Plasmodium berghei* in mice with the highest 73.95% parasitaemia suppression observed at a dose of 600 mg/kg/day [9]. The dichloromethane/methanol extracts of* A. ferox *and* A. maculate *showed antiplasmodial activity with an IC_50_ = 8 and 12.4 mg/mL, respectively [32].

The leaves latex of* A. percrassa* and compounds isolated from it, aloin A/B and microdontin A/B, have been investigated for their* in vivo* antimalarial activity in* P. berghei* infected mice. Both the latex and the isolated compounds possessed significant antimalarial activity with parasitemia suppression of 73.6% from latex treatment [22]. Similarly, the leaves latex of* A. pulcherrima *possesses* in vivo *antimalarial activity in mice infected with the rodent parasite* P. berghei*. Furthermore, a significant reduction of parasitemia was observed in groups treated with the isolated compounds, nataloin, and 7-hydroxyaloin, specifically at a dose of 200 mg/kg [24]. Homonataloin A/B has been isolated as a major component of the latex of* A. citrana* and both the latex and homonataloin A/B exhibited significant antimalarial activity in* P. berghei* infected mice [23].

Moreover,* in vitro* antimalarial test of ether leaves extracts of* A. dawei* using the chloroquine diphosphate as control has shown a potential inhibition of parasite growth against* P. falciparum *[33]. Similarly,* Aloe perryi *has been studied for its* in vitro *antiplasmodial activity and the result supports the use of the plant leaves latex in the treatment of malaria [34].* In vitro* antiplasmodial activity test of aqueous leaf extract and isolated compounds of* A. vera* collected from different climatic regions in India has been conducted. Different chemosuppressive effect of samples collected from different climatic area has been observed with EC_50_ values ranging from 0.289 to 1056 *μ*g/ml. Aloin and aloe-emodin, the compounds isolated from* A. vera* leaf extract, have been shown to possess dose-dependent antiplasmodial activity with EC_50_ value 67 *μ*g/ml and 22 *μ*g/ml, respectively. The standard drug, chloroquine, has been reported with 0.034 *μ*g/ml EC50 value [21].

Similarly in this study, both leaves latex of* A. megalacantha* and TLC isolates obtained from the latex revealed a significant dose-dependent chemosuppression activity against* P. berghei *in Swiss albino mice. The observed activity could be due to single or combined action of secondary metabolites found in the leaf latex. In this study, the individual isolates show better parasite growth inhibition when administered separately than the leaves latex. This could be due to the antagonism effect of either the isolates obtained or minor compounds found in the leaves latex which can lead to cancel in part the antimalarial activity of compounds.

According to Zeleke* et al*.,* in vivo* antimalarial activity can be classified as moderate, good, and very good if an extract showed percent growth inhibition ≥50% at a dose of 500, 250, and 100 mg/kg body weight/day, respectively [35]. Based on this, leaves latex of* A. megalacantha* had shown a moderate antiplasmodial activity against* P. berghei* infected mice.

Mean survival time (MST) of* P. berghei* infected mice is another parameter to evaluate an antimalarial activity of test substance. It has been reported that dose-dependent mean survival time of the mice treated with leaves latex and isolated compounds of* A. percrassa* was observed and has been reported as it was statistically significant as compared to the negative control group [22]. Mice treated with latex and compounds isolated from* A. pulcherrima* have been survived for longer time than the negative control group, even though it was not in a dose-dependent manner and not statistically significant as compared to negative control [24].

Similarly, the survival time of mice treated with either leaves latex or TLC isolates of* A. megalacantha *was observed in a dose-dependent phenomenon. This might happen due to its suppressive effect on the parasite growth. Even though the mean survival time of mice treated with latex, AM_1_ or AM_2_, at a dose of 100 mg/kg was not significant, they were survived for longer time relative to the negative control group. All mice treated with the latex and isolates at 200 and 400 mg/kg were shown a significant survival time (P<0.05) compared to vehicle treated mice.

The isolates obtained from leaves latex of* A. megalacantha* are believed to be anthraquinones and/or their oxidative derivatives because of having similar physical appearance and retention factor (R_f_) values on TLC with anthrones and other anthraquinones derivatives isolated from leaves latex of* A. harlana* [36],* A. percrassa* [22]*, A. citrina* [23]*, A. trigonantha* [37],* A. pulcherrima *[24]*, A. sinana *[38],* A. marlothii*, and* A. rupestris *[29]. It has been also reported that leaves of* Aloe* species are store house of anthraquinones and other metabolites [28]. This proposition is also supported by the report of Abdissa* et al*., who described the presence of anthraquinones and preanthraquinones like chrysophanol, aloesaponarin I, aloesaponarin II, and aloesaponarin III in ethyl acetate root extract of* A. megalacantha* [26].

Body weight loss and reduction in rectal temperature are the key characteristic of rodent malaria. Basir* et al.* have described weight loss as one of the dramatic manifestations of rodent malaria. This could be due to reduced food and water intake. They have reported that progression of complications like severe anemia and hypoglycemia could be another possible mechanism which leads to break down of lipids and proteins as a source of energy which can lead to weight loss [31]. Treatment of* P. berghei* infected mice with leaf latex and isolated compounds of* A. percrassa *[22],* A. citrina *[23], and* A. pulcherrima *[24] has shown dose-dependent weight loss reduction. Furthermore, significant reduction of colonic temperature has been also seen through infection of mice with* P. berghei* [31].

In the current study, weight loss and hypothermia were observed in the vehicle treated* P. berghei* infected mice. However, treatment with leaves latex and isolates of* A. megalacantha* was shown to reduce malaria caused weight loss and hypothermia. The isolate AM_3_ showed greater increase in weight as compared to chloroquine. Thus, the observed weight gain after treatment with leaves latex and isolates could be attributed to enhanced food intake of mice and/or inhibited pathophysiology of the disease. Hence, all the leaves latex and TLC isolates revealed preventive effect in reducing hypothermia with the highest effect observed in the isolates. The possible mechanism of prevention could be due to reduced debilitation of parasitemia as hypothermia is directly proportional to the parasitemia. Increased appetite could also be attributed to reduction in hypothermia by increasing body weight.

## 5. Conclusion

From the findings of the present study, a significant dose-dependent chemosuppression of both leaves latex and TLC isolates obtained from* A. megalacantha* was observed in* P. berghei* infected mice. Moreover, both the leaves latex and isolates could be considered safe at a single dose of 2000 mg/kg as they did not cause any manifestation of acute toxicity. The antimalarial activity of the leaves latex of this plant in part may be attributed to the presence of AM_1_, AM_2_, and AM_3_, which require further purification and elucidation of the structures of the isolated compound, could serve as the starting compound for the development of novel synthetic antimalarial drugs. Thus, results of the present study justify and may also support the traditional use of the plant as antimalarial agent.

## Figures and Tables

**Table 1:** Percentage suppression and mean survival time of *Plasmodium berghei* infected mice after treatment with leaf latex and TLC isolates obtained from *A. megalacantha*.

Test substance	Dose (mg/kg/day)	%Parasitemia ± SEM	%Suppression	MST (days)
NC	10 ml/kg	47.0 ± 3.49	0.00	6.4 ± 0.25
Latex	100	32.7 ± 1.21*∗∗*	30.4	7.2 ± 0.37
	200	26.6 ± 2.99*∗∗*	43.4	9.0 ± 0.51*∗*
	400	20.5 ± 1.22*∗∗*	56.4	10.1 ± 0.51*∗∗*
AM_1_	100	31.8 ± 5.05*∗∗*	32.3	7.6 ± 0.6
	200	22.9 ± 3.73*∗∗*	51.3	8.8 ± 0.58*∗*
	400	15.3 ± 3.78*∗∗*	67.4	10.0 ± 0.84*∗∗*
AM_2_	100	28.3 ± 5.90*∗∗*	39.8	7.8 ± 0.49
	200	23.2 ± 3.90*∗∗*	50.6	8.8 ± 0.37*∗*
	400	16.8 ± 4.19*∗∗*	64.2	9.6 ± 0.68*∗∗*
AM_3_	100	22.3 ± 4.77*∗∗*	52.6	8.2 ± 0.58*∗*
	200	14.4 ± 2.38*∗∗*	69.4	9.6 ± 0.55*∗∗*
	400	9.6 ± 1.75*∗∗*	79.6	10.4 ± 0.51*∗∗*
CQ	25	0.00*∗∗*	100	ND

Values are presented M ± SEM; n =5; *∗* mean value is significant (*P*<0.05) and *∗∗* mean value highly significant (P<0.001) when compared with negative control (NC) group; ND = no death within the follow-up period; MST= mean survival time in days.

**Table 2:** Body weight of *Plasmodium berghei* infected mice after administration of the leaf latex and TLC isolates of *Aloe megalacantha.*

Test substance	Dose (mg/kg/day)	Wt D_0_ (g)	Wt D_4_ (g)	Mean difference
NC	10 ml/kg	21.9 ± 0.63	20.9 ± 0.64	-1.0 (4.6)
Latex	100	23.2 ± 0.48	23.1 ± 0.44	-0.1 (0.4)
	200	21.4 ± 1.22	21.7 ± 1.01	0.3 (1.4)
	400	20.1 ± 0.23	21.2 ± 0.59	1.1 (5.5)
AM_1_	100	20.6 ± 0.38	20.8 ± 0.42	0.2 (1.0)
	200	20.7 ± 0.38	21.6 ± 0.32	0.9 (4.4)
	400	21.1 ± 0.35	22.4 ± 0.56	1.3 (6.2)
AM_2_	100	22.3 ± 0.91	22.7 ± 1.03	0.2 (0.9)
	200	21.7 ± 0.67	22.8 ± 0.78	1.1 (5.1)
	400	21.2 ± 0.45	22.7 ± 0.28	1.5 (7.1)
AM_3_	100	20.6 ± 0.48	21.8 ± 0.47	1.2 (5.8)
	200	21.9 ± 0.45	23.5 ± 0.88	1.6 (7.3)
	400	21.3 ± 0.46	23.3 ± 0.57	2.0 (9.4)
CQ	25	21.9 ± 0.38	23.2 ± 0.27	1.3 (5.9)

Values are presented as M ± SEM; n refers to number of mice, n = 5; Wt D_0_: weight before treatment on day zero; Wt D_4_: weight after treatment on fifth day (day 4); values in parenthesis indicate percent of change.

**Table 3:** Mean difference of rectal temperature of *P. berghei* infected mice after treatment with leaf latex and TLC isolates obtained from *A. megalacantha.*

Test substance	Dose (mg/kg/day)	T^0^ D_0_ (°C)	T^0^ D_4_ (°C)	Mean difference
NC	10 ml/kg	37.8 ± 0.31	34.6 ±0.33	-3.2 (8.5)
Latex	100	38.02 ± 0.22	35.66 ± 0.13*∗*	-2.36 (6.6)
	200	37.45 ± 0.26	35.82 ± 0.31*∗*	-1.63 (4.6)
	400	37.5 ± 0.28	36.16 ± 0.21*∗*	-1.34 (3.7)
AM_1_	100	37.7 ± 0.19	35.9 ± 0.29*∗*	-1.7 (4.5)
	200	37.6 ± 0.37	36.6 ± 0.22*∗*	-1.0 (2.7)
	400	37.7 ± 0.33	36.8 ± 0.27*∗*	-0.9 (2.4)
AM_2_	100	37.6 ± 0.30	36.1 ± 0.50*∗*	-1.5 (4.0)
	200	37.4 ± 0.28	36.6 ± 0.52*∗*	-0.8 (2.1)
	400	37.4 ± 0.26	36.7 ± 0.31*∗*	-0.7 (1.9)
AM_3_	100	37.6 ± 0.24	36.5 ± 0.17*∗*	-1.1 (2.9)
	200	37.6 ± 0.23	36.7 ± 0.17*∗*	-0.9 (2.4)
	400	37.8 ± 0.33	36.9 ± 0.26*∗*	-0.9 (2.4)
CQ	25	37.4 ± 0.32	37.7 ± 0.26*∗*	0.3 (0.8)

Values are presented as M ± SEM; n =5; *∗* mean value is significant (P<0.001) when compared with negative control group; T^0^ D_0_: temperature before treatment on day zero; T^0^ D_4_: temperature after treatment on fifth day (day 4); values in parenthesis indicate % of change.

## Data Availability

The datasets used and/or analyzed during the current study are available from the corresponding author on reasonable request.

## Abbreviations

EC_50_: Effective oncentration at 50%
EPHI: Ethiopian Public Health Institute
IC_50_: of a substance 50%
MST: Mean urvival ime
OECD: Organization for Economic Cooperation and Development
RBC: Red Blood Cells
TLC: Thin ayer hromatography
UV: Ultraviolet
WHO: World Health Organization.
